# Long-term compromised immune regulation after rituximab induction in blood group incompatible (ABOi) living-donor renal transplantation – 5 year results of a prospective pilot study

**DOI:** 10.3389/fimmu.2025.1706158

**Published:** 2025-12-12

**Authors:** Rolf Weimer, Hristos Karakizlis, Fabrice Renner, Hartmut Dietrich, Volker Daniel, Caner Süsal, Christian Schüttler, Daniel Kämper, Dominik Leicht, Michael Wörlen, Katrin Milchsack, Lene Renner, Maximilian Stich, Hermann-Josef Gröne, Andreas Hecker, Rüdiger Hörbelt, Winfried Padberg, Gerhard Opelz

**Affiliations:** 1 Department of Internal Medicine, University of Giessen, Giessen, Germany; 2 Institute of Immunology, University of Heidelberg, Heidelberg, Germany; 3 Transplant Immunology Research Center of Excellence TIREX, Koç University School of Medicine, Istanbul, Türkiye; 4 Institute of Medical Virology, University of Giessen, Giessen, Germany; 5 Medical Faculty, University of Heidelberg, Heidelberg, Germany; 6 Institute of Pharmacology, University of Marburg, Marburg, Germany; 7 Department of Surgery, University of Giessen, Giessen, Germany

**Keywords:** kidney transplantation, ABO incompatible, rituximab, B cell repopulation, protocol biopsies, acute rejection, severe infectious disease, HLA antibodies

## Abstract

**Background:**

An increased risk of severe infectious disease and acute antibody-mediated rejection (AMR) has been described after ABOi renal transplantation. We performed a prospective renal transplant study up to 5 years posttransplant to detect long-term immunological effects of rituximab administration.

**Methods:**

Mononuclear cell subsets in peripheral blood, regional lymph nodes and protocol biopsies, *in-vitro* T and B cell responses, serum sCD30 and neopterin were assessed in 85 renal transplant recipients (living donation: n=25 ABOi, n=30 ABO compatible (ABOc); deceased donation (DD): n=30, all ABO compatible), and IgG anti-HLA antibodies by single antigen assay in ABOc and ABOi patients.

**Results:**

An increased frequency of severe infectious diseases in ABOi recipients (doubled versus ABOc within 2 years, P = 0.042) coincided with profoundly downregulated peripheral blood B cell subsets for at least 2 years, impaired *in-vitro* B cell responses (T-dependent: 2 years, versus ABOc: P = 0.004) and significantly lower CD4+ and CD8+ T cell counts (versus ABOc; 6 months, P = 0.046 and 3 months, P = 0.011, respectively). In regional lymph nodes, we found a significant downregulation of naive B cells (P = 0.031) and short lived plasma cells (P<0.0005) at the time of transplantation. In protocol graft biopsies, rituximab induced B cell depletion at 3 months (P<0.001), but counter-regulatory enhanced counts of T cells (P = 0.041), macrophages (P = 0.021) and plasma cells (P = 0.033) at 1 year. This immune activation was associated with a temporary rise in neopterin levels (P ≤ 0.024 versus ABOc, day 14 until 1 year), CD4 helper activity (P = 0.019 versus ABOc at 2 years) and NK cell counts (P = 0.034 versus ABOc, 4 years; P ≤ 0.040 versus DD, 3–5 years), a missing impact of rituximab on sCD30 levels and HLA antibody formation, and an increased frequency of biopsy-proven acute rejection (3–12 months, P = 0.003) and AMR (P = 0.008 within 5 years).

**Conclusions:**

An increased frequency of severe infectious diseases in ABOi renal transplant recipients may be explained by rituximab-induced long-term immunological effects on CD4+ and CD8+ T cell counts and the prolonged depletion of B cell subsets together with compromised B cell responses. In protocol graft biopsies, rituximab induced early B cell depletion but counter-regulatory proinflammatory effects coinciding with an increased acute rejection frequency.

**Registration Details:**

ClinicalTrials.gov, identifier NCT01136395; EudraCT, identifier No.: 2009-012198-36.

## 1 Introduction

Rituximab has been proven to suppress anti-ABO titer rebound in ABOi renal transplantation. However, an increased risk of severe infectious disease (1–3) and AMR (2, 3) has been described after ABOi compared to ABOc renal transplantation in two meta-analyses (2, 3) and by CTS registry data (1), respectively. In both meta-analyses, AMR was more frequent in ABOi renal transplantation, whereas overall biopsy-proven acute rejection was found to be more frequent only by de Weerd and Betjes (2). CTS data showed a significantly but only slightly decreased ABOi patient survival in the first posttransplant year only, which was caused by early severe infectious complications (1). In the meta-analysis of de Weerd and Betjes (2), usually rituximab was used as induction in ABOi patients. Studies using splenectomy – known for increased rates of severe infectious complications - were excluded. They also found a small 1% decline of 1-year patient survival and a significantly increased rate of severe nonviral infections, slightly increased CMV viremia and conflicting results regarding BK viremia. Scurt et al. (3) found an increased mortality in ABOi patients up to 5 years posttransplant compared to ABOc patients, and in the rituximab treated subgroup of ABOi patients up to 3 years. Sepsis was more frequent in ABOi patients, whereas the rituximab treated ABOi subgroup did not show an increased risk of infectious complications, and only a trend for a higher risk of BK-virus associated nephropathy. A more recent meta-analysis (4) found rather high rates of infectious complications in 5 of the 15 extracted studies, but detailed data are lacking and rejection data are not provided.

Aim of this prospective pilot study is to assess short- and long-term effects of rituximab induction in ABOi renal transplant recipients and of living compared to deceased donation on immunological parameters of graft outcome and on viral replication. The focus of the current presentation is to detect short-term and especially long-term immunological effects of rituximab induction on graft outcome in ABOi renal transplantation. For this purpose, we analyzed clinically relevant immune parameters such as CD4 helper activity, *in-vitro* B cell responses, serum sCD30 and neopterin levels, HLA antibody formation and protocol biopsies in a prospective renal transplant study up to 5 years posttransplant.

## 2 Materials and methods

### 2.1 Patient groups

In this non-randomized, prospective, open pilot study (phase 2 study; off-label use of rituximab) 30 consecutive deceased brain-death donor (DD) renal transplant recipients (ABO blood group compatible (ABOc); allocation by Eurotransplant), 30 consecutive ABOc and 25 consecutive ABO incompatible (ABOi; with rituximab induction) living donor renal transplant recipients of the Giessen transplant center were included in the study between January 2010 and May 2014; follow-up was 5 years. Inclusion criteria were adult renal transplant recipients, first or retransplants, and informed study consent. Exclusion criteria were combined transplants of more than one organ and contraindications against study drugs. Primary endpoints were immune parameters relevant for transplant outcome (CD4 helper activity, *in-vitro* B cell responses, serum sCD30 and neopterin levels), HLA antibody formation and counts and distribution of immune cells in protocol biopsies. Secondary endpoints were patient and graft survival, graft function and proteinuria, incidence of acute rejections and of severe infectious diseases. Maintenance immunosuppressive (intention-to-treat) treatment consisted of tacrolimus (Tacr; Prograf^®^) with early switch to Tacr-MR (modified release; Advagraf^®^), enteric-coated mycophenolate sodium (Myfortic^®^) and prednisolone according to the Giessen protocol. In ABOi patients, maintenance immunosuppression was started one day before the first immunoadsorption (IA; blood group specific IA, in case of donor-specific HLA antibodies (DSA) blood group unspecific IA). IA was performed pretransplant usually every other day to reach a target anti-ABO titer of 1:8, and posttransplant only if anti-ABO titers exceeded 1:8 in the first and 1:16 in the second week posttransplant. Induction therapy consisted of rituximab (Mabthera^®^; 375mg/m^2^) in ABOi patients (2–4 weeks before transplantation according to the baseline anti-ABO titer; a second or third administration of the standard dosage was allowed if no complete B cell depletion (<8 B cells/µl of peripheral blood) was achieved or the target anti-ABO titer could otherwise not be reached) and generally of basiliximab (Simulect^®^). Instead of basiliximab, prophylactic rabbit ATG (rATG; Fresenius, Oberursel, Germany; 9mg/kg preoperatively, 3mg/kg days 1–3 according to CD3 counts) was given in patients with maximum panel reactive antibodies (PRA) ≥20% or retransplantation. 10g IVIG was given in DD and ABOc patients pretransplant and on days 1,2 and 4 posttransplant. In ABOi patients, 0.5g/kg IVIG was given 4 days pretransplant, after testing of anti-ABO titers in 3 different IVIG preparations.

The patient groups were comparable with respect to most of the clinical features and transplant data including donor and recipient age, immunization events, induction therapy (with the exception of rituximab administration; **Table 1**), maintenance immunosuppression (**Table 1**), renal diseases (P = 0.143; data not shown; low incidence of diabetic nephropathy (n=7/85 (8%))), and pretransplant cytomegalovirus (CMV) serostatus of donors and recipients (P = 0.107; data not shown). As expected, preemptive transplants were more frequent and waiting times were shorter in living versus DD renal transplant recipients (with differences between ABOi and ABOc patients not reaching statistical significance; **Table 1**), as were shorter cold ischemia times and the worse HLA matching compared to DD patients.

**Table 1:** Clinical features and transplant data of patients who received a renal graft from a living donor (ABOc, blood group compatible; ABOi, blood group incompatible) and from a deceased donor (DD; blood group compatible), respectively.

	ABOc	ABOi	DD	P^1^
Number of patients	30^2^	25^2^	30	
Recipient age (years)	45±3	50±2	52±1	0.118
RRT (% HD/PD/PR)^3^	18(60%)-2(7%)-10(33%)	19(76%)-3(12%)-3(12%)	26(87%)-4(13%)-0(0%)	0.005
Waiting time (months)^4^	12±3	24±5	78±6	<0.001
Number of retransplants	2 (7%)	2 (8%)	2 (7%)	0.899
PRA max^5^≥20%	2 (7%)	1 (4%)	3 (10%)	0.870
PRA max^5^	3±1	3±2	5±2	0.005
PRA rec^6^	1±1	0±0	2±1	0.224
Blood transfusions	15 (50%)	14 (56%)	13 (43%)	0.682
Number of blood transfusions	1.6±0.4	1.8±0.4	1.3±0.4	0.510
Female patients	9 (30%)	6 (24%)	11 (37%)	0.638
Pregnancies	5 (17%)	4 (16%)	10 (33%)	0.223
Number of pregnancies	0.3±0.1	0.4±0.2	0.9±0.3	0.160
Donor age (years)	51±2	51±2	45±3	0.289
HLA A,B,DR mismatches	3.6±0.3	4.0±0.3	2.3±0.3	<0.001
Cold ischemia time (hours)	1.7±0.1	2.0±0.1	13.2±0.9	<0.001
Induction immunosuppressive treatment				
rATG induction	2 (7%)	2 (8%)	5 (17%)	0.458
Basiliximab induction	28 (93%)	22 (88%)	25 (83%)	0.490
Rituximab induction^7^	2 (7%)	25 (100%)	1 (3%)	<0.001
Maintenance immunosuppressive treatment^8^				
1 year posttransplant^8^				
T/Myc - T/Aza - T/Le -	90%-3%-7%-	68%-14%-14%-	80%-7%-7%-	0.694
T/ERL - ERL – T	0%-0%-0%	5%-0%-0%	3%-0%-3%	
Tacr trough level [ng/ml]	7.2±0.4	6.6±0.5	6.5±0.4	0.698
Myc dosage [mg/day]	907±50	804±82	938±45	0.259
Prednisolone dosage [mg/day]	5.1±0.5	6.7±1.3	5.8±0.6	0.594
2 years posttransplant^8^				
T/Myc - T/Aza - T/Le -	83%-3%-14%-	67%-10%-10%-	83%-7%-7%-	0.306
T/ERL - ERL-T	0%-0%-0%	14%-0%-0%	3%-0%-0%	
Tacr trough level [ng/ml]	6.4±0.3	5.6±0.4	5.9±0.4	0.182
Myc dosage [mg/day]	893±50	810±82	835±60	0.564
Prednisolone dosage [mg/day]	2.7±0.5	3.7±1.0	3.1±0.6	0.679
5 years posttransplant^8^				
T/Myc - T/Aza - T/Le -	69%-8%-19%-	72%-6%-6%-	78%-7%-4%-	0.296
T/ERL - ERL - T	0%-4%-0%	17%-0%-0%	7%-0%-4%	
Tacr trough level [ng/ml]	6.3±0.3	5.7±0.3	6.1±0.4	0.355
Myc dosage [mg/day]	850±56	734±66	843±52	0.434
Prednisolone dosage [mg/day]	2.0±1.1	1.4±0.5	1.6±0.8	0.337

^1^Kruskall-Wallis H test, Mann-Whitney U test (for comparison of 2 patient groups), Chi-square test and Fisher´s exact test, respectively, were used for statistical comparison. ^2^One patient was succesfully desensitized for ABOi living-donor renal transplantation but transplantation could not be performed because of a posterior reversible encephalopathy syndrome (drop-out). In the ABOi group one further drop-out occurred due to refusal of a further study participation at 44 months posttransplant (clinical data included till study completion), and one drop out occurred in the ABOc group at 12 months (move to England). ^3^RRT= renal replacement therapy (percentage of patients treated with hemodialysis (HD), peritoneal dialysis (PD) and preemptive transplantation (PR), respectively). Preemptive transplants were more frequent in ABOc versus ABOi patients without reaching statistical significance (P=0.060). ^4^Waiting time was longer in ABOi versus ABOc patients without reaching statistical significance (P=0.058). ^5^PRA max, ^6^PRA rec= maximum and recent panel reactive antibodies pretransplant, respectively; DD patients showed significantly increased PRA max (P=0.003 vs. ABOc, P=0.020 vs. ABOi) without differences between ABOc and ABOi patients (P=0.740). However, differences were clinically not relevant as shown by the low and between the patient groups comparable percentage of patients with PRA max≥20%. This was also true looking at PRA max>10% (P=0.697; 3 (10%), ABOc; 1 (4%), ABOi; 3 (10%), DD). ^7^Pretransplant rituximab induction was given in all ABOi patients according to the study protocol, and in 2/30 ABOc and 1/30 DD patients because of donor-specific HLA antibodies (protocol violation). ^8^Initial maintenance immunosuppressive treatment consisted of tacrolimus (T), enteric-coated mycophenolate sodium (Myc) and prednisolone in all patients; Statistical comparisons between two of the three patient groups were also not significant with regard to immunosuppressive regimens, tacrolimus trough levels, daily dosage of Myc and prednisolone; T=tacrolimus; Myc=enteric-coated mycophenolate sodium; Aza=azathioprine; Le=leflunomide; ERL=everolimus.

All patients at high CMV risk (CMV-IgG positive donor and negative recipient; rATG treatment) received valganciclovir prophylaxis for 6 months. Oral cotrimoxazole and pentamidine inhalation, respectively, was used in all patients for pneumocystis jiroveci prophylaxis (9 months). Rituximab treated patients received fluconazole prophylaxis (14 days posttransplant) in addition, and an antiviral prophylaxis with valaciclovir (3 months) and valganciclovir in high CMV risk patients (6 months), respectively. Immunological tests were performed pretransplant (in ABOi recipients also before rituximab administration), 14 days, 3 and 6 months and 1, 2, 3, 4 and 5 years posttransplant.

### 2.2 Flow cytometric analysis of peripheral blood mononuclear cells

PBMC subsets and their expression of costimulatory ligands were determined by double- and triple-fluorescence (triple fluorescence for the indicated B cell subsets) and laser flow-cytometry as described previously (5, 6). The following monoclonal antibodies (MoAb) were used: anti-CD4-FITC, anti-CD4-PE, anti-CD4-PE-Cy7, anti-CD8-FITC, anti-CD14-FITC, anti-CD19-FITC, anti-CD20-FITC, anti-CD25-PE, anti-CD28-PE and anti-CD40-PE (Becton Dickinson (BD), Heidelberg, Germany). Mouse IgG1-PE (BD) was used as isotype control. For detection of B cell subsets, anti-CD19-FITC/anti-CD27-PE/anti-CD20-PE-Cy7, anti-CD19-FITC/anti-CD22-PE/anti-CD20-PE-Cy7 and anti-CD19-FITC/anti-CD138-PE/anti-CD38-PE-Cy7 were used together with anti-CD19-FITC/mouse IgG1-PE/mouse IgG1-PE-Cy7 as isotype controls (all antibodies from BD).

Simultest IMK-Lymphocyte (BD) reagents and software were used to detect granulocytes, PBMC, monocytes (CD14+), lymphocytes, NK cells (CD3-CD16+ and/or CD56+), CD3+ T cells, CD4+ T cells, CD8+ T cells and CD19+ B cells in peripheral blood. Absolute cell counts were assessed using BD Trucount tubes (BD) according to the manufacturer´s protocol.

FoxP3+ Tregs (FoxP3+ CD25+ CD4 cells) in peripheral blood were determined essentially according to the manufacturer´s protocol (lysed whole blood cells were used instead of isolated PBMC) using the following MoAbs: anti-human FoxP3-PE (clone 259D/C7; BD), anti-human CD25-PE-Cy7 (clone M-A251, BD) and anti-human CD4-FITC (clone RPA-T4; BD). Mouse IgG1-PE and mouse IgG1-PE-Cy7 (BD) were used as isotype controls, and anti-CD4-FITC, anti-CD4-PE and anti-CD4-PE-Cy7 (BD) for compensation of the flow cytometer. The Human FoxP3 Buffer Set (BD) was applied to fixation and permeabilization of lysed whole blood cells, staining of the given cell surface markers and intracellular staining of Human FoxP3. BD CellWash (BD) with 2% fetal calf serum was used as a modified staining buffer. Tregs (CD25high CD127low CD4 cells) in peripheral blood were determined using the following MoAbs: anti-CD4-FITC (BD)/anti-CD25-PE (BD)/anti-CD127-PE-Cy7 (eBioscience, Thermo Fisher Scientific GmBH, Munich, Germany) and anti-CD4-FITC/mouse IgG1-PE/mouse IgG1-PE-Cy7 (BD) as isotype controls.

Measurements were performed using a FACSCalibur flow cytometer (BD). Exclusion of dead cells was done by forward and side-scatter gating. 100,000 mononuclear cells were typically acquired for analysis. Results are given in % of gated cells and molecules of equivalent phycoerythrin (MEPE) for indicated parameters (those showing better discrimination from isotype controls by MEPE), respectively. MEPE describes the molecule density per cell and was calculated by comparing staining of samples with a standard curve. Individual standard curves were established for each assessment by analysis of a mixture of 8 calibrated bead populations containing 8 different MEPE levels per bead (Sphero Rainbow Calibration Particles, 8 peaks, 3.0-3.4 µm; BD). The results were calculated by subtracting the respective isotype controls results.

### 2.3 Serum sCD30 and neopterin

Sera were thawed and tested by ELISA for sCD30 (Bender MedSystems, Vienna, Austria) and neopterin (Brahms, Berlin, Germany). Neopterin values were corrected for graft function by dividing values by the serum creatinine (Neopterin/S-Cr, given in nmol/g S-Cr (5)).

### 2.4 Flow cytometric analysis of iliac lymph nodes

One regional (iliac) lymph node - removed during transplant surgery - was dissected with scissors and scalpel, rinsed with staining buffer (BD CellWash (BD) with 2% fetal calf serum (BD)) and passed through a 40µm sieve. After washing, the resulting cell suspension (10^7^/ml) was stained as described above for PBMC (Simultest IMK-Lymphocyte kit (BD); staining of B cell subsets and Tregs (CD25high CD127low CD4 cells)).

### 2.5 PWM-stimulated allogeneic cocultures and SAC I-stimulated B cell cultures

T-cell dependent B cell responses of PWM-stimulated allogeneic cocultures of patient B and control T cells, T-independent B cell responses of SAC I-stimulated B cell cultures and helper and suppressor activity of whole T cells and CD4+ T cells, respectively, were determined as described previously (5–7).

### 2.6 IgG anti-HLA antibodies

IgG anti-HLA antibodies were assessed on a Luminex basis in ABOc and ABOi recipients pretransplant and 3 and 6 months, and 1, 2, 3, 4 and 5 years posttransplant. LABScreen™ Mixed Class I & II (One Lambda, West Hills, CA, US) was used for screening, and single antigen assays were used in case of positive screening results (LABScreen™ Single Antigen Class I, LABScreen™ Single Antigen Class II; One Lambda). This analysis was not done in DD recipients, as the full HLA donor typing (9 loci) was not available.

### 2.7 Immunohistochemistry of protocol biopsies

Protocol biopsies were generally recommended 3 and 12 months posttransplant and were performed at 3 months in 58 of 83 patients with functioning grafts (70%; 22/30 (73%), ABOc; 15/23 (65%), ABOi; 21/30 (70%), DD) and at 12 months in 34 of 82 (41%; 14/30 (47%), ABOc; 9/22 (41%), ABOi; 11/30 (37%), DD) patients. Immunohistochemistry was performed as described previously (8). For immunostaining, we used primary antibodies against CD3 (monoclonal rabbit anti-CD3, ab135372, clone SP162, 1:200; Abcam, Cambridge, UK), CD19 (monoclonal rabbit anti-CD19, ab134114, clone EPR5906, 1:250; Abcam), CD20 (monoclonal mouse anti-CD20cy, M0755, clone L26, 1:200; Dako/Agilent), CD38 (monoclonal rabbit anti-CD38, ab183326, clone SP149, 1:100; Abcam), CD68 (monoclonal mouse anti-CD68, MO876, clone PG-M1, 1:100; Dako/Agilent), CD138 (monoclonal mouse anti-CD138, ab128936, clone EPR 6454, 1:700; Abcam), FoxP3 (monoclonal mouse anti-FoxP3, ab20034, clone 236A/E7, 1:50; Abcam) and SV40 T-antigen (monoclonal mouse anti-SV40T, clone PAb416, 1:200 Millipore/Merck, Darmstadt, Germany), respectively. Visualization was performed using the Dako REAL Detection System (alkaline phosphatase/red for rabbit and mouse antibodies; K5005; Dako/Agilent).

Biopsy samples were scanned using a Hamamatsu Scanner (Nanozoomer 2.0 RS, Hamamatsu Photonics, Japan). Manual evaluation of the data files was performed using the NDP.view 2.7.39 software (free edition). For quantitative analysis, results were given in cells per mm^2^ of biopsy area. Semiquantitative estimation of distribution patterns was analyzed for CD3+ T cells, FoxP3+ Treg, CD19+ B cells and CD20+ B cells (given in % of biopsies with missing or very rare occurrence versus diffuse infiltration versus focal infiltration versus nodular infiltration) and for plasma cells (% of biopsies with missing versus scattered occurrence versus focal infiltration).

### 2.8 Virological screening

BK and JC virus screening was performed by an in-house real time PCR (9) with hybridization probes in serum and urine specimens pretransplant (serum testing only), 3 and 6 months, and 1,2,3,4 and 5 years posttransplant. JC viremia was not detected. CMV PCR (Artus CMV LC PCR Kit, No. 4503065, Qiagen, Hilden, Germany) screening was performed in serum at the same time points.

### 2.9 Statistics

Data are presented as means and SEM. Wilcoxon signed-rank test, Mann-Whitney U test, Kruskall-Wallis H test and Chi-square or Fisher`s exact test were used for statistical analysis. A 0.05 significance level was defined for univariate testing.

## 3 Results

### 3.1 Patient and graft outcome

We found no significant differences in patient and graft survival between the three patient groups, although no patient death occurred in ABOc patients in contrast to 3 deaths in ABOi and also in DD patients during the 5-year posttransplant follow-up (details given in **Table 2**). 5-year death-censored graft survival, graft function and proteinuria were also comparable between the patient groups (**Table 2**), whereas 1-year graft function (but not proteinuria, P = 0.370) was significantly different (ClCr: 74 ± 4 ml/min, ABOc; 62 ± 5 ml/min, ABOi; 56 ± 5 ml/min, DD; P = 0.031; ABOc versus DD, P = 0.011; other comparisons between two patient groups: not significant). We found no significant differences between the measured creatinine clearance values (mean of two calculations) of ABOc and ABOi living kidney donors (116 ± 2 ml/min, ABOc versus 117 ± 4 ml/min, ABOi; P = 0.660).

**Table 2:** Five year outcome in the ABOc and ABOi living-donor and the deceased donor (DD) patient groups.

	ABOc	ABOi	DD	P^1^
Number of patients	30^2^	25^2^	30	
Posttransplant ARF^3^	0% (0/30)	4% (1/24)	27% (8/30)	0.001
AR^4^ incidence 0-5 years	17% (5/29)	38% (9/24)	17% (5/30)	0.130
AR 0-1 year	10% (3/30)	33% (8/24)	13% (4/30)	0.067
AR 0-3 months	7% (2/30)	13% (3/24)	13% (4/30)	0.743
AR >3-12 months	3% (1/30)	26% (6/23)	0% (0/30)	0.002
AR >1-2 years	3% (1/29)	9% (2/22)	3% (1/30)	0.672
AR >2-3 years	0% (0/29)	0% (0/22)	0% (0/30)	1.000
AR >3-5 years	3% (1/29)	0% (0/22)	0% (0/30)	0.630
AR^4^ incidence 0-5 years	17% (5/29)	38% (9/24)	17% (5/30)	0.130
-Rituximab treated ABOc/DD excluded^5^	12% (3/25)	38% (9/24)	11% (3/27)	0.047
AIR^4^ incidence 0-5 years	14% (4/29)	25% (6/24)	13% (4/30)	0.539
-Rituximab treated ABOc/DD excluded^5^	12% (3/25)	25% (6/24)	11% (3/27)	0.360
Acute AMR^4^ incidence 0-5 years	3% (1/29)	17% (4/24)	7% (2/30)	0.278
-Rituximab treated ABOc/DD excluded^5^	0% (0/25)	17% (4/24)	0% (0/27)	0.008
Patient survival	100% (29/29)^6^	88% (21/24)^6^	90% (27/30)^6^	0.149
Graft survival	90% (26/29)^6^	79% (19/24)^6^	90% (27/30)^6^	0.550
Death-censored graft survival	90% (26/29)^6^	83% (19/23)^6^	93% (27/29)^6^	0.610
Graft function^7^				
S-Cr (mg/dl)^7^	1.4±0.1	1.6±0.2	1.6±0.2	0.535
ClCr (ml/min)^7^	72±7	59±7	60±5	0.275
Proteinuria (mg/day)^8^	126±17	124±16	177±60	0.547
Severe infectious disease^9^				
0-2 years	20% (6/30)^9^	46% (11/24)^9^	27% (8/30)	0.117
0-5 years	33% (10/30)	50% (12/24)	33% (10/30)	0.369
Types of infectious diseases				
Gastrointestinal infections	n=2	n=2	n=0	
Respiratory infections	n=2	n=1	n=2	
Skin infections	n=1	n=1	n=0	
Urinary tract infections	n=5	n=17	n=5	
Urosepsis	n=3^10^	n=14^10^	n=5^10^	
Wound infections	n=1	n=0	n=1	
Viral infections				
Chicken pox	n=1	n=0	n=0	
CMV infection	n=3	n=2	n=3	
HSV 1/2 infection	n=0	n=1	n=0	
Herpes zoster	n=0	n=0	n=1	
CMV disease^11^				
0-2 years	17% (5/30)	4% (1/23)	17% (5/30)	0.368
0-5 years	20% (6/30)	9% (2/23)	17% (5/30)	0.645
Severe CMV disease^11^				
0-2 years	10% (3/30)	4% (1/23)	10% (3/30)	0.785
0-5 years	10% (3/30)	9% (2/23)	10% (3/30)	1.000
BKV infection				
BK viremia				
0-2 years	13% (4/30)	26% (6/23)	17% (5/30)	0.514
0-5 years	20% (6/30)	26% (6/23)	17% (5/30)	0.699
BK nephropathy^12^				
0-2 years	7% (2/30)	4% (1/23)	7% (2/30)	1.000
0-5 years	7% (2/30)	4% (1/23)	7% (2/30)	1.000

^1^Kruskall-Wallis H test, Mann-Whitney U test (for comparison of 2 patient groups), Chi-square test and Fisher´s exact test, respectively, were used for statistical comparison. ^2^One patient was succesfully desensitized for ABOi living-donor renal transplantation but transplantation could not be performed because of a posterior reversible encephalopathy syndrome (drop-out). In the ABOi group one further drop-out occurred due to refusal of a further study participation at 44 months posttransplant (clinical data included till study completion), and one drop out occurred in the ABOc group at 12 months (move to England). The two drop outs at 12 and 44 months posttransplant, respectively, were included in the analysis of infectious complications. Two ABOi patients died 3 and 10 months posttrransplant, respectively; as they suffered from severe infectious diseases they were included in the respective analysis, but the patient who died at the 3 month posttransplant time-point was not included in the BKV and CMV evaluations because of too short follow-up (i.e. less than 6 months). ^3^Post-transplant acute renal failure (ARF) was defined as need to perform at least one post-transplant hemodialysis procedure in the first week posttransplant. ^4^Acute rejection (AR), acute interstitial (cellular) rejection (AIR) and acute antibody-mediated rejection (AMR) incidence; acute AMR occurred in the first posttransplant year only; anti-ABO antibody induced acute AMR occurred in one ABOi patient only (day 3 posttransplant); ^5^Rituximab was given in all ABOi patients as induction therapy according to the study protocol (in two patients also posttransplant within the first two weeks for anti-ABO titer rebound and anti-ABO induced acute AMR, respectively; in two more patients, for acute AMR at 1 month and for chronic AMR at 15 months, respectively), and in 4/30 ABOc patients (two patients pretransplant because of donor-specific HLA antibodies, one patient at month 4 because of acute AMR, one patient because of PTLD starting month 43 for 6 times overall) and in 3/30 DD patients (one highly immunized patient pretransplant and at month 1 because of combined acute AMR with acute interstitial rejection (AIR); one patient because of acute AMR at month 5 and one because of donor specific antibody formation at month 2; protocol violation). ^6^In the *ABOi group*, one patient died 3 months posttransplant due to a malignant antiphospholipid antibody syndrome, one patient 10 months posttransplant due to a sigmadiverticulitis with perforation, followed by profound wound healing disorders - including insufficiency of the descendostoma anastomosis - and sepsis with multiorgan failure, and one patient due to intracerebral bleeding and tentorial herniation 44 months posttransplant; in the first two ABOi patients, sepsis induced graft loss occurred; None of these 3 ABOi patients received rATG induction. Two more graft losses in ABOi patients resulted from a legionella sepsis at 48 months and from treatment resistant (including eculizumab) acute and chronic antibody-mediated rejection after a 4th renal transplantation at 17 months, respectively. In the *DD group*, 3 patients died due to heart failure (sudden cardiac death) at month 33, 40 and 44, respectively, with preceding graft loss in 2 of these 3 patients (due to BK-virus associated nephropathy at month 38 and renal artery stenosis at month 40, respectively). None of the *ABOc patients* died, but graft loss occurred in 3 patients (due to recurrence of membranoproliferative glomerulonephritis (MPGN) I in the graft at month 40 and 48, respectively, and in one patient due to non-adherence with acute and chronic interstitial rejection at month 44). ^7^S-Cr = serum creatinine; ClCr = measured creatinine clearance; ^8^22 of 26 ABOc patients, 12 of 19 ABOi patients and 17 of 27 DD patients (only patients with functioning grafts considered), respectively, were investigated; ^9^Severe infectious disease was defined as need for in-hospital treatment; Comparison of ABOi with ABOc patients 2 years posttransplant shows a significant difference in the incidence of severe infectious disease (P=0.042); follow-up data after graft loss were not available; ^10^Seven episodes of urosepsis and two of acute cystitis (with multiresistant bacteria) occurred in one ABOi patient, 4 episodes of urosepsis in another ABOi patient and 2 episodes of urosepsis in one DD patient (no rituximab treatment), so that the number of patients with urosepsis did not differ significantly between the three patient groups (ABOc, n=3/29 (10.3%); ABOi, n=5/24 (20.8%); DD, n=4/30 (13.3%); P=0.586), and also not between rituximab treated versus not rituximab treated patients (6/31 (19.4%) versus 6/52 (11.5%), P=0.350). ^11^The pretransplant CMV serostatus was not significantly different between the patient groups (P=0.107); ^12^BK-virus associated nephropathy was confirmed by transplant biopsy.

These data imply a surprisingly good outcome in the DD patient group, which, perhaps importantly, showed comparable recipient and donor age and also immunization events, but better HLA compatibility compared to ABOc and ABOi patients (**Table 1**). Only one DD patient was transplanted via the Eurotransplant senior program.

Clinical outcome data regarding acute rejection and infectious complications are remarkable despite the relatively low patient numbers in each of the patient groups. Although usually acute rejections occur more frequently in the first three months posttransplant, we found a significantly increased frequency of acute rejections between 3 and 12 months posttransplant in ABOi compared to ABOc and DD patients (6/23 (26%), ABOi; 1/30 (3%), ABOc; 0/30 (0%), DD; P = 0.002; ABOi versus ABOc, P = 0.034; ABOi versus DD, P = 0.004), but no differences in other posttransplant periods (**Table 2**). Overall, the 1-year acute rejection incidence was increased in ABOi versus ABOc patients (8/24 (33%) versus 3/30 (10%), P = 0.046), whereas statistical comparison of all three patient groups did not reach significance (P = 0.067, **Table 2**). When rituximab treated ABOc and DD patients were excluded (n=4 (2 pretransplant administrations), ABOc; n=3 (1 pretransplant), DD; details see **Table 2**), 5-year acute AMR but not AIR incidence was significantly increased in ABOi patients (P = 0.008; **Table 2**). It has to be considered, however, that pretransplant rituximab was given in 3 non-ABOi patients because of an increased rejection risk due to donor-specific HLA antibodies.

#### 3.1.1 Infectious complications

Noteworthy, severe infectious diseases occurred twice often in ABOi compared to ABOc patients in the first two years posttransplant but not beyond (P=0.042; **Table 2**). Urinary tract infection (especially urosepsis) was the most frequent severe infectious disease. Types of severe infections did not differ significantly between the groups (**Table 2**) and also not between overall rituximab treated versus non-treated patients (data given for urosepsis in **Table 2**). CMV disease was numerically less frequent in ABOi compared to ABOc and DD patients (**Table 2**), possibly due to antiviral prophylaxis performed in all ABOi patients. BK viremia was somewhat more frequent in ABOi compared to ABOc and DD patients, but differences did not reach statistical significance (**Table 2**). The incidence of BK-virus associated nephropathy was less than 10% in all patient groups.

### 3.2 PBMC subsets, costimulatory ligand expression and Tregs

The increased risk of severe infectious complications within the first 2 years posttransplant coincided with lower peripheral blood T cell counts in ABOi versus ABOc recipients up to 6 months (CD4+ T cells: 3 months, P = 0.025; 6 months, P = 0.046; CD8+ T cells: 3 months, P = 0.011; 6 months, not significant P = 0.095; **Table 3**), whereas no significant differences were found between ABOi and DD patients. CD25 (IL-2R alpha) and CD28 costimulatory ligand expression on T cells was comparable between ABOi and ABOc patients but significantly different compared with DD patients (lower CD25 expression on CD4+ T cells (P = 0.013, 3 months); increased CD28 expression on CD4+ T cells (P = 0.001, 1 year; P = 0.018, 2 years; P = 0.032, 5 years) and CD8+ T cells (P = 0.006, 1 year; P = 0.056, 2 years; P = 0.013, 5 years); **Table 3**). FoxP3+ Treg counts were slightly, but significantly lower in DD patients at 1 and 2 years posttransplant (1 year, P = 0.028 versus ABOc; 2 years, P = 0.043 versus ABOi; **Table 3**) without significant differences between ABOc and ABOi patients. However, CD25high CD127 low Treg showed no significant differences between the 3 patient groups (**Table 3**). Pretransplant rituximab induction showed no impact on Tregs, as shown by comparison of Treg levels in ABOi patients before ritxumab induction with pretransplant levels (21 ± 2 days after administration; before start of other immunosuppressive drugs). Interestingly, NK cell counts were significantly increased in ABOi versus ABOc recipients at 4 years (P = 0.034) and versus DD patients 3–5 years posttransplant (P = 0.017, 3 years; P = 0.024, 4 years; P = 0.040, 5 years), whereas NK cell counts of ABOc and DD patients were comparable (**Table 3**).

### 3.3 B cell repopulation after rituximab administration

**Figure 1:**
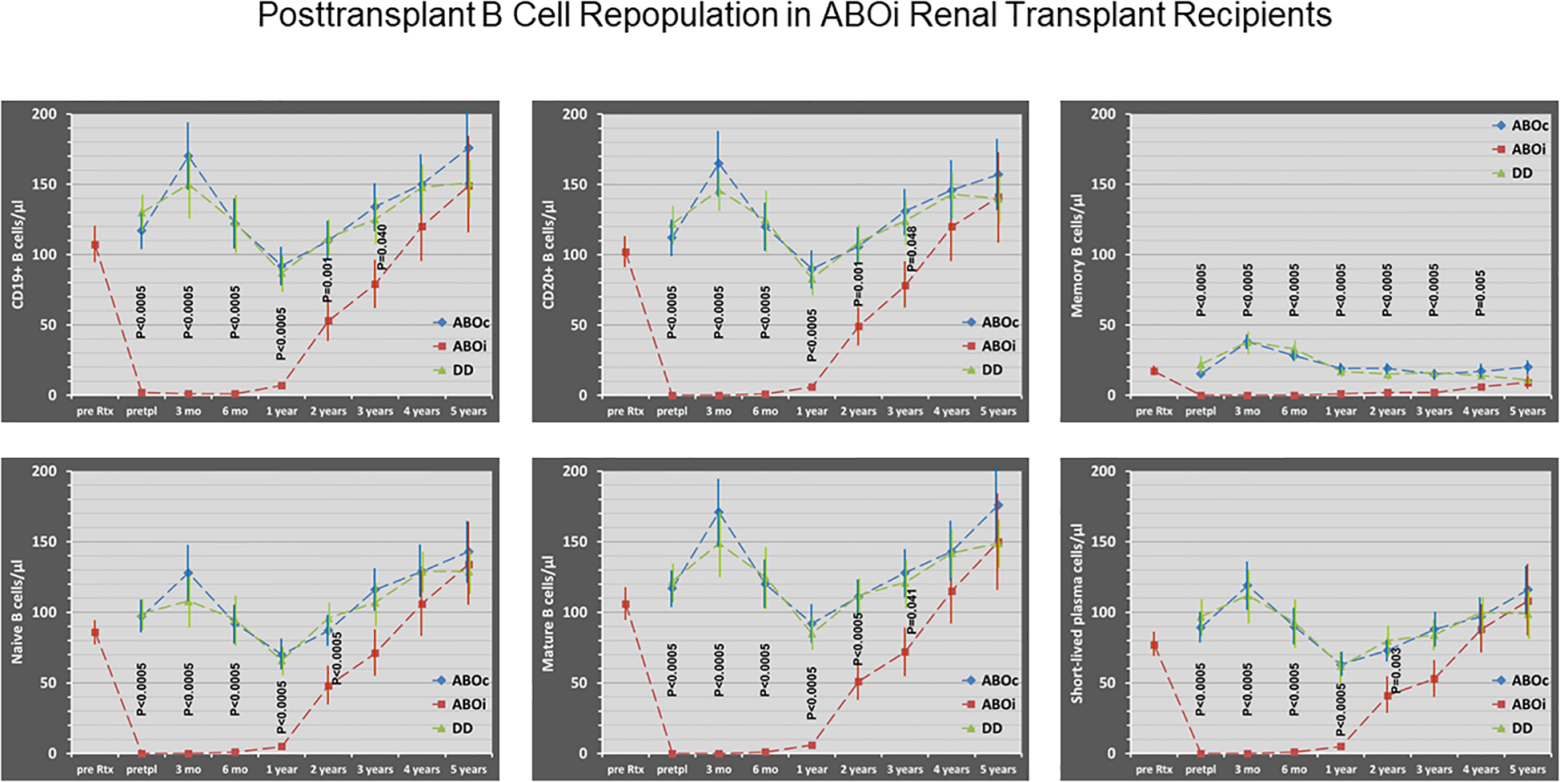
Peripheral blood B cell subsets (CD19+ B cells, CD20+ B cells, memory B cells (CD27+ CD19+ cells), naive B cells (CD27- CD19+ cells), mature B cells (CD22+ CD19+ cells) and short lived plasma cells (CD19+ CD38+ CD138- cells)) in ABO incompatible (ABOi; red line) and ABO compatible (ABOc; blue line) living donor renal transplant recipients, and in DD renal transplant recipients (DD; green line) before rituximab administration (pre Rtx; in ABOi patients), pretransplant (pretpl; before start of any other immunosuppressive treatment), 3 und 6 months, and 1,2,3,4 and 5 years postttransplant. Long lived plasma cells (CD19- CD38+ CD138+ cells) were only occasionally found (mean frequencies of 1±0 up to 2±1 cells/μl) in all patient groups and were therefore not included in the diagram. Mean and SEM are indicated. P values are given for statistically significant differences between the patient groups (Kruskall-Wallis H test). After rituximab induction in ABOi recipients, all of the presented pretransplant B cell subset counts (obtained before start of any other immunosuppressive treatment) showed a significant decline compared to the counts before rituximab administration (P<0.0005). This resulted in profoundly downregulated peripheral blood B cell subset counts in ABOi recipients compared to ABOc and DD patients for at least 2-3 years, most pronounced with regard to memory B cells (even 4 years).

After rituximab induction in ABOi recipients, all of the presented B cell subsets in peripheral blood declined significantly before start of any other immunosuppressive treatment. B cells were virtually absent up to 1 year posttransplant and the profound downregulation of the B cell subsets lasted at least 2–3 years, most pronounced with regard to memory B cells (for 4 years; **Figure 1**). At 2 years posttransplant, most of the B cell subsets in ABOi patients reached about 50% of the counts in ABOc or DD patients (**Figure 1**), whereas memory B cells were scarcely found (19 ± 4/µl, ABOc; 2 ± 0/µl, ABOi; 15 ± 3/µl, DD; P<0.0005). At 3 years posttransplant, ABOi patients still showed reduced counts of naive B cells (P = 0.025 versus ABOc), mature B cells (P = 0.028 versus ABOc, P = 0.023 versus DD) and short lived plasma cells (P = 0.023 versus ABOc, P = 0.053 versus DD). Interestingly, B cell CD40 costimulatory ligand expression in ABOi patients was significantly downregulated 1 year and 2 years posttransplant (no analysis performed earlier due to low B cell counts) compared to DD patients (P = 0.003, 1 year; P = 0.024, 2 years; **Table 3**) without significant differences between ABOc and ABOi patients. ABOc patients also showed reduced B cell CD40 expression compared to DD patients 1 to 5 years posttransplant (3 months, P = 0.454; 1 year, P = 0.020; 2 years, P = 0.004; 5 years, P = 0.041) thereby excluding an impact of rituximab on the downregulated CD40 expression. In ABOi patients, B cell CD25 expression was slightly reduced only at 1 year posttransplant (P = 0.036 versus ABOc; **Table 3**).

### 3.4 CD4 helper activity, *in-vitro* B cell responses

**Figure 2:**
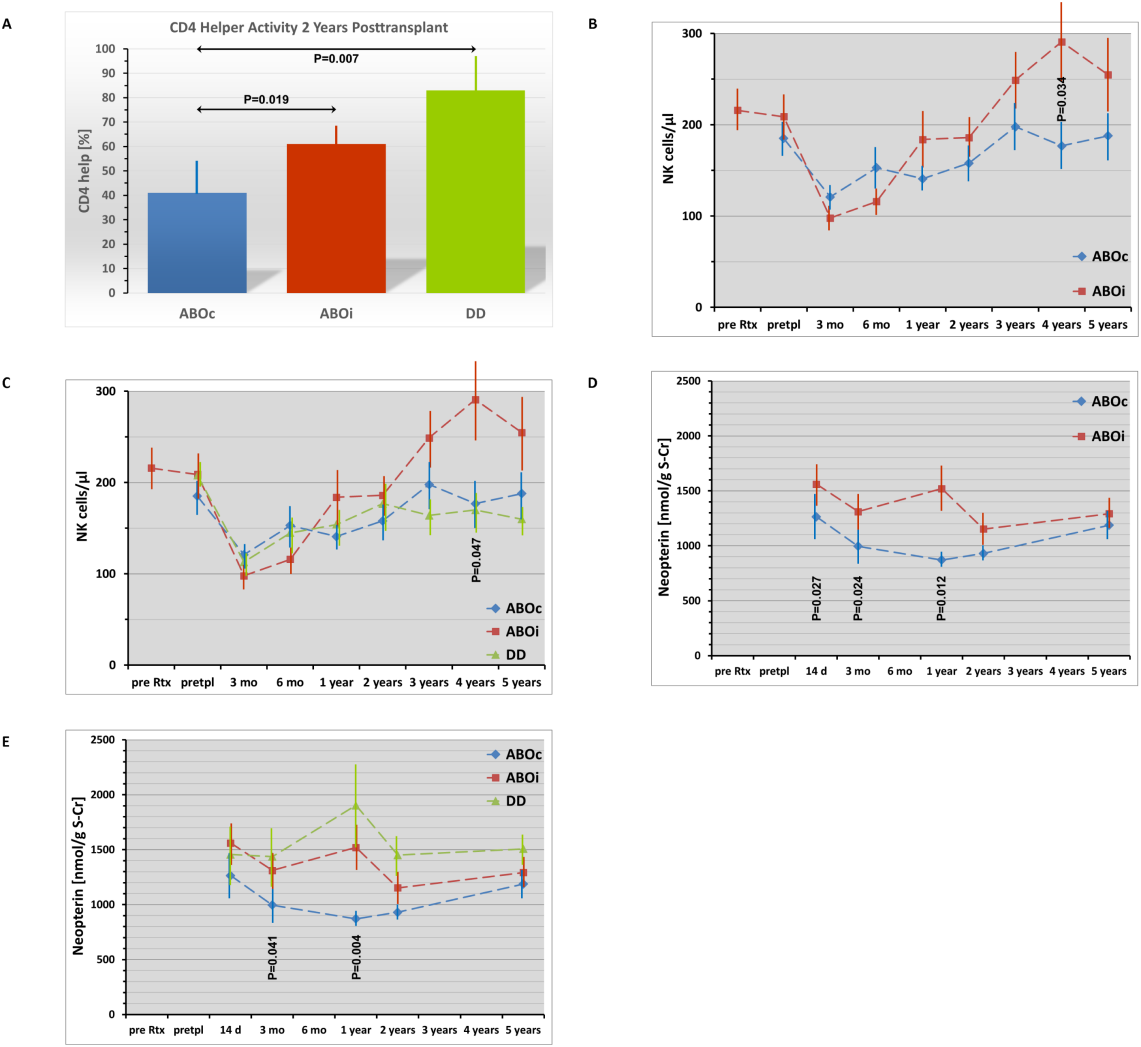
Temporary immune activation in ABO incompatible (ABOi; marked in red) compared to ABO compatible (ABOc; marked in blue) living donor renal transplant recipients shown for CD4 helper activity at 2 years posttransplant **(A)** and for peripheral blood NK cell counts **(B, C)** and serum neopterin **(D, E)** during the 5-year posttransplant follow-up (pre Rtx: before rituximab administration in ABOi patients; pretpl: pretransplant, before start of any other immunosuppressive treatment). Mean and SEM are indicated. P values are given for statistically significant differences between the patient groups. Kruskall-Wallis H Test was used for statistical comparison of the three patient groups, Mann-Whitney U Test (Wilcoxon rank-sum test) for statistical comparison between 2 of the 3 patient groups. The numerically increased *CD4 helper activity* in ABOi versus ABOc patients did not reach statistical significance at 1 year (P=0.077) but did at 2 years (P=0.019; **Figure 2A**) followed by comparable helper activity at 5 years (P=0.228; see table 3). *Peripheral blood NK cell counts* significantly increased at 4 years in ABOi compared to ABOc patients (**Figure 2B**) and were elevated compared to DD patients at 3 (P=0.017), 4 (P=0.024) and 5 years (P=0.040; overall significant differences between the 3 patient groups only at 4 years, see **Figure 2C**). *Serum neopterin levels* showed a temporary increase in ABOi compared to ABOc patients at 14 days up to 1 year posttransplant (**Figure 2D**) indicating an increased activation of the T cell / monocyte system in this period, whereas DD patients demonstrated significantly elevated serum neopterin levels compared to ABOc recipients 3 months up to 5 years posttransplant (P=0.042, 3 months; P=0.002, 1 year; P=0.038, 2 years; P=0.035, 5 years; **Figure 2E**) without significant differences between DD and ABOi patients during the complete 5-year follow-up.

The prolonged downregulation of B cell subsets after rituximab induction in ABOi recipients coincided with impaired *in-vitro* B cell responses up to 2 years posttransplant (T-dependent: 3 months, P = 0.041 vs. ABOc, P = 0.021 vs. DD; 1 year, not significant (P = 0.111 vs. ABOc, P = 0.075 vs. DD); 2 years, P = 0.004 vs. ABOc, P = 0.012 vs. DD; T-independent: 3 months and 1 year, not significant; 2 years, P = 0.053 vs. ABOc, P = 0.657 vs. DD; **Table 3**). CD4 helper and suppressor activity was comparable between the patient groups with the exception of the 2 year posttransplant time point (lower CD4 helper function in ABOc vs. ABOi (P = 0.019) and DD patients (P = 0.007); lower CD4 suppressor function in DD vs. ABOi (P = 0.034) and ABOc patients (P = 0.007; **Table 3** and **Figure 2**).

**Table 3:** Peripheral blood granulocytes and mononuclear cell subsets (PBMC), expression of costimulatory ligands, helper and suppressor activity of whole T cells and CD4+ T cells, in-vitro T cell-independent and T cell-dependent B cell responses (immunoglobulin-secreting cell (ISC) formation) and serum sCD30 and neopterin levels in living-donor ABO-compatible (ABOc), ABO-incompatible (ABOi) and deceased-donor ABO-compatible (DD) renal transplant recipients before rituximab administration (in ABOi patients), pretransplant and at different time points up to 5 years posttransplant.

	Before Rituximab				Pretransplant			
	ABOc	ABOi	DD	P^1^	ABOc	ABOi	DD	P^2^
Granulocytes/µl	-	4403±287^3^	-	0.006	4968±394	5553±454	5392±382	0.510
Mononuclear cells/µl	-	1808±75	-	0.136	1984±134	1780±154	1871±64	0.310
Monocytes/µl	-	376±30	-	0.107	425±37	437±43	432±33	0.809
Lymphocytes/µl	-	1432±76	-	0.009	1559±116	1343±132	1439±61	0.189
NK cells /µl	-	216±23	-	0.693	185±18	209±24	208±14	0.445
T cells /µl	-	1019±64	-	0.330	1177±103	1049±126	1009±62	0.379
T helper activity [%]	-	73±14	-	0.614	84±11	56±11	77±14	0.264
T suppressor activity [%]	-	9±16	-	0.287	20±11	27±12	-4±21	0.976
**CD4+ T cells /µl**	**-**	**700±50**	**-**	**0.648**	**776±59**	**753±97**	**658±39**	**0.296**
CD4 helper activity [%]	-	57±13	-	0.527	67±9	59±11	74±14	0.722
CD4 suppressor activity [%]	-	10±11	-	0.394	11±12	13±14	-2±18	0.874
CD25+ CD4 cells [%]	-	49.0±2.5	-	0.097	49.8±2.4	50.6±2.5	56.4±1.9	0.070
CD28+ CD4 cells [%]	-	97.0±1.0	-	0.862	96.8±1.0	97.4±0.7	94.1±1.5	0.215
FoxP3+ Treg [%]^5^	-	6.7±0.4	-	0.445	7.4±0.5	7.1±0.4	6.5±0.4	0.322
CD127 low Treg [%]^5^	-	8.1±0.4	-	0.494	8.4±0.5	7.9±0.5	8.0±0.3	0.721
**CD8+ T cells /µl**	**-**	**329±29**	**-**	**0.171**	**405±61**	**316±40**	**365±33**	**0.458**
CD25+ CD8 cells [%]	-	5.1±0.9	-	0.196	6.5±1.1	5.7±1.0	7.5±1.3	0.445
CD28+ CD8 cells [%]	-	66.4±4.6	-	0.031	72.7±4.0	65.7±4.5	65.5±3.5	0.258
**CD19+ B cells /µl**	**-**	**107±13**	**-**	**<0.0005**	**117±13**	**2±1**	**130±13**	**<0.0005**
T-independent B cell response [ISC/10^6^ B cells]^6^	-	2705±820	-	-	834±296	-^7^	3161±1688	0.573
T-dependent B cell response [%]	-	66±12	-	-	54±11	-^7^	55±14	0.089
CD25+ B cells [MEPE]	-	53±9	-	-	63±9	-^7^	75±20	0.529
CD40+ B cells [MEPE]	-	860±73	-	-	877±59	-^7^	997±66	0.124
**sCD30 [ng/ml]**	**-**	**69±6**	**-**	**0.254**	**69±5**	**67±5**	**65±5**	**0.675**
**Neopterin [nmol/g S-Cr]**	**-**	**ND**	**-**	**-**	**ND**	**ND**	**ND**	**-**
	14 Days				3 Months			
	ABOc	ABOi	DD	P	ABOc	ABOi	DD	P
**Granulocytes/µl**	**ND^4^**	**ND**	**ND**	**-**	**4821±404**	**4443±413**	**6102±480**	**0.034**
**Mononuclear cells/µl**	**ND**	**ND**	**ND**	**-**	**2548±188**	**1889±158**	**1986±125**	**0.045**
**Monocytes/µl**	**ND**	**ND**	**ND**	**-**	**439±37**	**551±61**	**525±58**	**0.282**
**Lymphocytes/µl**	**ND**	**ND**	**ND**	**-**	**2109±168**	**1338±142**	**1461±107**	**0.003**
**NK cells /µl**	**ND**	**ND**	**ND**	**-**	**121±13**	**98±13**	**113±12**	**0.430**
**T cells /µl**	**ND**	**ND**	**ND**	**-**	**1694±156**	**1143±135**	**1106±96**	**0.009**
T helper activity [%]	ND	ND	ND	-	49±6	69±13	87±12	0.077
T suppressor activity [%]	ND	ND	ND	-	33±7	-15±21	19±10	0.264
**CD4+ T cells /µl**	**ND**	**ND**	**ND**	**-**	**1256±123**	**833±112**	**705±67**	**0.003**
CD4 helper activity [%]	ND	ND	ND	-	47±6	52±14	64±11	0.438
CD4 suppressor activity [%]	ND	ND	ND	-	50±5	-4±21	31±8	0.058
CD25+ CD4 cells [%]	ND	ND	ND	-	53.0±2.5	57.0±4.1	63.6±2.2	0.013
CD28+ CD4 cells [%]	ND	ND	ND	-	98.1±0.6	98.3±0.5	94.8±1.5	0.162
FoxP3+ Treg [%]^4^	ND	ND	ND	-	5.5±0.4	5.9±0.5	5.1±0.4	0.339
CD127 low Treg [%]^4^	ND	ND	ND	-	7.4±0.4	6.7±0.6	7.2±0.5	0.725
**CD8+ T cells /µl**	**ND**	**ND**	**ND**	**-**	**485±50**	**304±31**	**422±44**	**0.046**
CD25+ CD8 cells [%]	ND	ND	ND	-	6.0±1.0	6.2±1.6	8.4±1.5	0.209
CD28+ CD8 cells [%]	ND	ND	ND	-	75.8±3.9	69.0±4.9	66.7±3.9	0.206
**CD19+ B cells /µl**	**ND**	**ND**	**ND**	**-**	**170±24**	**1±0**	**150±23**	**<0.0005**
T-independent B cell response [ISC/10^6^ B cells]^6^	ND	ND	ND	-	5519±1752	145±84^8^	1392±349	0.123
T-dependent B cell response [%]	ND	ND	ND	**-**	72±14	0±0	50±10	0.061
CD25+ B cells [MEPE]	ND	ND	ND	**-**	78±13	-^7^	99±38	0.602
CD40+ B cells [MEPE]	ND	ND	ND	**-**	1251±80	-^7^	1231±104	0.454
**sCD30 [ng/ml] **	**15±2**	**17±2**	**17±2**	**0.820**	**22±2**	**24±3**	**25±2**	**0.703**
**Neopterin [nmol/g S-Cr]**	**1265±199**	**1561±192**	**1457±269**	**0.086**	**996±146**	**1311±164**	**1438±267**	**0.041**
	6 Months				1 Year			
	ABOc	ABOi	DD	P	ABOc	ABOi	DD	P
**Granulocytes/µl**	**4805±537**	**3893±601**	**4422±449**	**0.395**	**4597±412**	**4121±367**	**5083±408**	**0.278**
**Mononuclear cells/µl**	**2494±197**	**1861±175**	**2196±160**	**0.110**	**2290±137**	**1985±165**	**2067±132**	**0.251**
**Monocytes/µl**	**531±42**	**530±60**	**519±45**	**0.897**	**518±35**	**537±42**	**539±34**	**0.986**
**Lymphocytes/µl**	**1964±188**	**1331±140**	**1677±144**	**0.078**	**1772±132**	**1448±150**	**1529±120**	**0.151**
**NK cells /µl**	**153±24**	**116±15**	**145±19**	**0.763**	**141±13**	**184±31**	**154±19**	**0.560**
**T cells /µl**	**1641±154**	**1122±128**	**1301±129**	**0.058**	**1454±123**	**1165±136**	**1216±115**	**0.187**
T helper activity [%]	ND	ND	ND	-	66±11	89±13	56±12	0.080
T suppressor activity [%]	ND	ND	ND	-	9±16	2±19	35±13	0.100
**CD4+ T cells /µl**	**1150±121**	**780±116**	**773±74**	**0.043**	**1000±95**	**773±103**	**642±67**	**0.016**
CD4 helper activity [%]	ND	ND	ND	-	57±12	83±14	62±12	0.188
CD4 suppressor activity [%]	ND	ND	ND	-	31±11	-2±18	5±16	0.318
CD25+ CD4 cells [%]	53.6±2.4	58.2±2.6	60.4±2.4	0.123	51.3±2.5	55.4±3.1	54.4±2.2	0.345
CD28+ CD4 cells [%]	ND	ND	ND	-	97.8±0.7	97.5±0.8	92.3±1.8	0.001
FoxP3+ Treg [%]^4^	ND	ND	ND	-	5.8±0.4	5.3±0.4	4.7±0.3	0.093
CD127 low Treg [%]^4^	7.5±0.4	6.7±0.4	6.6±0.5	0.307	6.4±0.4	6.4±0.5	6.1±0.4	0.863
**CD8+ T cells /µl**	**489±60**	**339±45**	**543±75**	**0.145**	**440±59**	**393±46**	**576±68**	**0.169**
CD25+ CD8 cells [%]	6.0±1.0	6.2±1.3	7.8±1.5	0.563	5.5±0.8	5.2±1.2	5.5±1.3	0.548
CD28+ CD8 cells [%]	ND	ND	ND	-	73.6±3.9	63.6±5.5	51.8±4.4	0.006
**CD19+ B cells /µl**	**122±18**	**1±1**	**123±21**	**<0.0005**	**92±14**	**7±2**	**87±13**	**<0.0005**
T-independent B cell response [ISC/10^6^ B cells]^6^	ND	ND	ND	-	752±257	287±113^8^	2192±810	0.155
T-dependent B cell response [%]	ND	ND	ND	-	49±12	21±14^8^	61±12	0.080
CD25+ B cells [MEPE]	ND	ND	ND	-	70±11	33±5	100±48	0.146
CD40+ B cells [MEPE]	ND	ND	ND	-	1472±478	815±108	1405±108	0.004
**sCD30 [ng/ml]**	23±3	37±7	27±3	0.153	28±3	40±8	43±6	0.056
**Neopterin [nmol/g S-Cr]**	ND	ND	ND	-	871±63	1520±209	1904±372	0.004
	2 Years				3 Years			
	ABOc	ABOi	DD	P	ABOc	ABOi	DD	P
**Granulocytes/µl**	**4330±451**	**4884±370**	**4474±259**	**0.161**	**4932±492**	**5187±459**	**5218±321**	**0.338**
**Mononuclear cells/µl**	**2212±162**	**1934±152**	**2127±156**	**0.541**	**2324±172**	**2119±165**	**2137±128**	**0.842**
**Monocytes/µl**	**515±55**	**485±34**	**484±30**	**0.940**	**545±50**	**532±32**	**530±36**	**0.868**
**Lymphocytes/µl**	**1698±128**	**1449±128**	**1643±143**	**0.420**	**1779±143**	**1587±144**	**1607±118**	**0.704**
**NK cells /µl**	**158±20**	**186±22**	**177±26**	**0.513**	**198±26**	**249±31**	**164±19**	**0.070**
**T cells /µl**	**1343±114**	**1132±115**	**1292±129**	**0.438**	**1351±126**	**1167±117**	**1209±108**	**0.732**
T helper activity [%]	61±10	74±10	78±14	0.492	ND	ND	ND	-
T suppressor activity [%]	6±14	29±11	-9±17	0.306	ND	ND	ND	-
**CD4+ T cells /µl**	**875±75**	**712±77**	**673±66**	**0.081**	**843±73**	**744±103**	**639±49**	**0.124**
CD4 helper activity [%]	41±9	61±7	83±12	0.009	ND	ND	ND	-
CD4 suppressor activity [%]	22±13	17±13	-32±17	0.016	ND	ND	ND	-
CD25+ CD4 cells [%]	46.8±2.9	49.9±2.8	47.1±2.0	0.680	41.5±2.5	47.1±2.8	47.3±2.2	0.126
CD28+ CD4 cells [%]	97.5±0.7	96.3±1.1	92.6±1.7	0.018	ND	ND	ND	-
FoxP3+ Treg [%]^4^	5.5±0.5	5.7±0.4	4.8±0.3	0.158	ND	ND	ND	-
CD127 low Treg [%]^4^	5.4±0.5	5.3±0.7	5.0±0.3	0.835	5.3±0.4	5.8±0.4	5.4±0.3	0.673
**CD8+ T cells /µl**	**455±61**	**409±70**	**616±89**	**0.148**	**481±75**	**408±40**	**556±75**	**0.456**
CD25+ CD8 cells [%]	4.8±0.9	5.2±1.2	4.8±1.3	0.990	4.7±0.9	4.2±1.1	4.9±1.3	0.658
CD28+ CD8 cells [%]	65.6±4.4	59.9±6.0	49.3±4.3	0.056	ND	ND	ND	-
**CD19+ B cells /µl**	**110±14**	**53±15**	**112±13**	**<0.001**	**134±17**	**79±17**	**125±16**	**0.040**
T-independent B cell response [ISC/10^6^ B cells]^6^	1346±415	93±418	918±41^8^	0.137	ND	ND	ND	-
T-dependent B cell response [%]	48±10	10±3^8^	51±12	0.010	ND	ND	ND	-
CD25+ B cells [MEPE]	46±8	29±4	108±73	0.810	ND	ND	ND	-
CD40+ B cells [MEPE]	776±56	815±85	1048±62	0.008	ND	ND	ND	-
**sCD30 [ng/ml]**	36±4	30±4	57±12	0.001	41±3	38±4	42±3	0.401
**Neopterin [nmol/g S-Cr]**	931±70	1153±142	1450±178	0.111	ND	ND	ND	-
	4 Years				5 Years			
	ABOc	ABOi	DD	P	ABOc	ABOi	DD	P
**Granulocytes/µl**	**4521±421**	**4900±284**	**5238±314**	**0.088**	**4441±354**	**5019±435**	**5227±368**	**0.308**
**Mononuclear cells/µl**	**2180±156**	**2223±170**	**2140±139**	**0.986**	**2191±171**	**1960±131**	**2101±132**	**0.728**
**Monocytes/µl**	**515±48**	**515±51**	**508±37**	**0.900**	**456±37**	**455±38**	**487±39**	**0.827**
**Lymphocytes/µl**	**1665±125**	**1708±142**	**1632±127**	**0.872**	**1735±144**	**1505±123**	**1614±113**	**0.555**
**NK cells /µl**	**177±26**	**291±44**	**170±22**	**0.047**	**188±26**	**255±40**	**160±16**	**0.154**
**T cells /µl**	**1254±106**	**1174±111**	**1223±122**	**0.814**	**1293±118**	**1019±88**	**1230±107**	**0.225**
T helper activity [%]	ND	ND	ND	-	74±13	105±14	69±10	0.107
T suppressor activity [%]	ND	ND	ND	-	9±20	-1±12	23±8	0.154
**CD4+ T cells /µl**	**810±64**	**771±91**	**641±53**	**0.056**	**845±68**	**685±78**	**685±54**	**0.141**
CD4 helper activity [%]	ND	ND	ND	-	75±13	99±15	56±11	0.089
CD4 suppressor activity [%]	ND	ND	ND	-	7±18	3±19	19±13	0.892
CD25+ CD4 cells [%]	42.8±2.8	40.6±2.9	41.1±2.4	0.788	41.4±3.6	50.1±3.1	43.0±1.9	0.126
CD28+ CD4 cells [%]	ND	ND	ND	-	97.7±0.8	96.2±1.3	93.7±1.5	0.032
FoxP3+ Treg [%]^4^	ND	ND	ND	-	6.3±0.6	6.0±0.6	6.0±0.3	0.960
CD127 low Treg [%]^4^	5.5±0.4	5.3±0.6	4.9±0.3	0.608	5.6±0.4	6.0±0.5	4.9±0.2	0.295
**CD8+ T cells /µl**	**424±63**	**399±49**	**555±84**	**0.299**	**430±67**	**330±28**	**529±70**	**0.107**
CD25+ CD8 cells [%]	5.5±1.2	4.0±0.9	5.0±1.6	0.671	5.1±1.0	4.6±1.1	4.8±1.4	0.896
CD28+ CD8 cells [%]	ND	ND	ND	-	72.2±5.2	59.3±5.7	50.9±4.0	0.013
**CD19+ B cells /µl**	**150±21**	**120±24**	**146±17**	**0.408**	**176±27**	**149±34**	**151±17**	**0.550**
T-independent B cell response [ISC/10^6^ B cells]^6^	ND	ND	ND	-	1607±561	1724±472	1210±477	0.174
T-dependent B cell response [%]	ND	ND	ND	-	60±15	22±74	47±10	0.198
CD25+ B cells [MEPE]	ND	ND	ND	-	41±7	37±9	31±5	0.710
CD40+ B cells [MEPE]	ND	ND	ND	-	530±64	652±92	647±41	0.158
**sCD30 [ng/ml]**	43±4	40±5	46±3	0.183	45±5	41±4	45±4	0.852
**Neopterin [nmol/g S-Cr]**	ND	ND	ND	-	1188±129	1292±134	1507±136	0.105

Mean and SEM are given; ^1^Wilcoxon signed-rank test was used for comparison of test results before rituximab administration with pretransplant results obtained before start of any other immunosuppressive treatment in ABOi patients; ^2^Kruskall-Wallis H Test was used for statistical comparison of the three patient groups; Mann-Whitney U Test (Wilcoxon rank-sum test) was used for statistical comparison, when only two patient groups were available for comparison; ^3^Data of mononuclear cell subsets are given as mean ± SEM of absolute cell counts, molecules of equivalent phycoerythrin (MEPE) and % of gated cells, respectively; ^4^ND = not done; ^5^FoxP3+ Treg = FoxP3+ CD25+ CD4 cells; CD127 low Treg = CD25high CD127low CD4 cells; ^6^ISC = immunoglobulin-secreting cells; ^7^no B cells or too low B cell numbers for this analysis; ^8^T-independent B cell responses were not significantly different between ABOi and ABOc or DD patients at 3 months and 1 year (3 months, P=0.121 vs. ABOc, P=0.457 vs. DD (due to low B cell counts, only 4 patients could be analyzed at 3 months); 1 year, P=0.399 vs. ABOc, P=0.075 vs. DD); 2 years, P=0.053 vs. ABOc, P=0.657 vs. DD; T-dependent B cell reponses were lower up to 2 years posttransplant: 3 months, P=0.041 vs. ABOc, P=0.021 vs. DD (due to low B cell counts, only 3 patients could be analyzed at 3 months); 1 year, not significant (P=0.111 vs. ABOc, P=0.075 vs. DD); 2 years, P=0.004 vs. ABOc, P=0.012 vs. DD.

### 3.5 Serum sCD30 and neopterin

In ABOi patients, comparison of serum sCD30 levels before rituximab administration with pretransplant levels (21 ± 2 days later; before start of other immunosuppressive drugs) showed no effect of rituximab an sCD30 levels (P = 0.254; **Table 3**). Differences of sCD30 levels between the patient groups were found only 1 year (increased levels in DD versus ABOc patients, P = 0.016; **Table 3**) and 2 years posttransplant (increased levels in DD vs. ABOc and ABOi patients; P = 0.010 and P<0.001, respectively; **Table 3**). Serum neopterin (corrected for graft function) was increased in ABOi versus ABOc patients 14 days posttransplant (P = 0.027; **Figure 2** and **Table 3**) and in both ABOi and DD patient groups compared to ABOc recipients 3 months (P = 0.024 and P = 0.042, respectively) and 1 year posttransplant (P = 0.012 and P = 0.002, respectively; **Table 3**). At 2 and 5 years, levels of ABOi and ABOc patients were comparable, whereas DD patients showed elevated levels compared to ABOc patients (P = 0.038 and P = 0.035, respectively; **Table 3**).

### 3.6 Flow cytometric analysis of regional lymph nodes

At the time of transplantation, we found a significant rituximab-induced downregulation of CD20+ but not CD19+ B cells (P<0.0005), of naive B cells (P = 0.031) and short lived plasma cells (P<0.0005; **Table 4**). Thus, the overall rate of B cells appeared not to be reduced after rituximab induction. The low percentage of CD20+ but not of CD19+ B cells may be explained by prevention of CD20 MoAb binding by rituximab coverage of the B cells.

**Table 4:** Flow cytometric analysis of regional lymph nodes at the time of transplantation in living-donor ABO-compatible (ABOc), ABO-incompatible (ABOi) and deceased-donor ABO-compatible (DD) renal transplant recipients.

	ABOc	ABOi	DD	P^1^
Number of patients	24	17	14	
% NK cells	2.2±0.2	1.8±0.2	2.7±0.4	0.101
% T cells	61.4±2.5	53.2±3.8	55.6±3.6	0.215
% CD4+ T cells	50.9±2.3	44.2±3.2	46.0±2.6	0.353
% CD25+	8.2±2.7	11.2±4.2	12.1±5.4	0.852
% Treg2	4.4±1.3	6.3±2.4	5.1±2.2	0.801
% CD8+ T cells	12.1±1.4	10.3±1.0	9.6±0.7	0.572
% CD19+ B cells	32.4±2.3	38.0±4.1	36.0±3.2	0.547
% CD20+ B cells	31.8±2.8	3.8±2.7	35.0±2.8	<0.0005
% Memory B cells^2^	25.6±2.7	33.8±3.9	27.3±2.8	0.213
% Naive B cells^2^	6.9±1.2	3.9±0.9	8.7±1.8	0.031
% Mature B cells^2^	32.5±2.3	39.0±4.2	35.9±3.2	0.499
% Short lived plasma cells^2^	16.5±1.5	8.7±1.6	18.1±2.3	<0.0005
% Long lived plasma cells^2^	0.0±0.0	0.0±0.0	0.0±0.0	0.241

Mean and SEM are given; ^1^Kruskall-Wallis H Test was used for statistical comparison of the three patient groups; Mann-Whitney U Test (Wilcoxon rank-sum test) was used for statistical comparison of two patient groups (data not given in the table): Significant differences in this analysis were found for CD20+ B cells (ABOc vs. ABOi, P<0.0005; ABOi vs. DD, P<0.0005), naive B cells (ABOc vs. ABOi, P=0.047; ABOi vs. DD, P=0.015) and short lived plasma cells (ABOc vs. ABOi, P<0.0005; ABOi vs. DD, P=0.001). ^2^ Treg = CD25high CD127low CD4 cells; Memory B cells = CD27+ CD19+ cells; Naive B cells = CD27- CD19+ cells; Mature B cells = CD22+ CD19+ cells; Short lived plasma cells = CD19+ CD38+ CD138- cells; Long lived plasma cells = CD19- CD38+ CD138+ cells.

### 3.7 Protocol biopsies

Protocol biopsies were performed in 70% and 41% of patients with functioning grafts at the 3 and 12 month posttransplant time point, respectively (see methods section). Cell subset analysis in these biopsies showed rituximab-induced B cell depletion in ABOi patients at 3 months (P<0.001 vs. ABOc and DD), but comparable B cell counts and even enhanced counts of CD3+ T cells (P = 0.041; ABOi vs. ABOc, P = 0.030; ABOi vs. DD, P = 0.050), CD68+ macrophages (P = 0.021; ABOi vs. ABOc, P = 0.008; ABOi vs. DD, P = 0.051; without a difference in the rate of macrophage infiltrated glomeruli) and CD138+ plasma cells (P = 0.033; ABOi vs. ABOc, P = 0.164 (not significant); ABOi vs. DD, P = 0.043) at 1 year (**Table 5**). Interestingly, in 2 of 14 (14%) ABOi patients diffuse or focal B cell infiltration was found at 3 months despite rituximab induction, and even nodular infiltration with CD19+ B cells in 3 of 8 (38%) ABOi patients at 1 year together with diffuse or focal plasma cell infiltration in 5 of 7 (71%) patients (**Table 5**). Treg counts and their pattern of distribution were not significantly different between the patient groups.

**Table 5:** CD3+ T cells, FoxP3+ regulatory cells, CD68+ macrophages, CD19+ B cells, CD20+ B cells and CD138+ plasma cells in 3 month and 1 year protocol biopsies of living-donor ABO-compatible (ABOc), ABO-incompatible (ABOi) and deceased-donor ABO-compatible (DD) renal transplant recipients.

	3 Months				1 Year			
	ABOc	ABOi	DD	P^1^	ABOc	ABOi	DD	P^1^
Number of biopsies^2^	22	15	21		14	9	11	
Cell counts per biopsy area:								
CD3+ T cells [per mm^2^]	104±28^3^	201±52	179±47	0.157	145±62	315±69	127±21	0.041
FoxP3+ cells [per mm^2^]	4±1	10±3	9±4	0.271	5±2	11±4	2±0	0.141
CD68+ macrophages [per mm^2^]^4^	101±31	142±49	112±19	0.244	87±30	294±73	132±41	0.021
CD19+ B cells [per mm^2^]	44±21	1±0	18±7	<0.001	17±7	12±7	11±4	0.932
CD20+ B cells [per mm^2^]	43±23	1±0	26±8	<0.001	12±4	9±3	16±3	0.330
CD138+ plasma cells [per mm^2^]	5±5	1±0	0±0	0.572	1±0	8±6	0±0	0.033
Pattern of distribution^5^								
CD3+ T cells	11%-22%-56%-11% (2/18) (4/18) (10/18) (2/18)	7%-27%-33%-33% (1/15) (4/15) (5/15) (5/15)	15%-30%-30%-25% (3/20) (6/20) (6/20) (5/20)	0.657	36%-14%-43%-7% (5/14) (2/14) (6/14) (1/14)	0%-14%-43%-43%(0/7) (1/7) (3/7) (3/7)	11%-33%-56%-0%(1/9) (3/9) (5/9) (0/9)	0.081
FoxP3+ cells	40%-25%-35%- 0%(8/20) (5/20) (7/20) (0/20)	13%-33%-47%-7%(2/15) (5/15) (7/15) (1/15)	37%-21%-32%-11%(7/19) (4/19) (6/19) (2/19)	0.536	46%- 8%-39%- 8% (6/13) (1/13) (5/13) (1/13)	29%-29%-29%-14%(2/7) (2/7) (2/7) (1/7)	33%-11%-56%- 0%(3/9) (1/9) (5/9) (0/9)	0.758
CD19+ B cells	26%-32%-21%-21%(5/19) (6/19) (4/19) (4/19)	86%- 7%- 7%- 0%(12/14) (1/14) (1/14) (0/14)	26%-37%-21%-16%(5/19) (7/19) (4/19) (3/19)	0.015	50%-17%-17%-17%(6/12) (2/12) (2/12) (2/12)	25%-38%- 0%-38% (2/8) (3/8) (0/8) (3/8)	25%-25%-50%- 0%(2/8) (2/8) (4/8) (0/8)	0.127
CD20+ B cells	14%-32%-36%-18% (3/22) (7/22) (8/22) (4/22)	86%-14%- 0%- 0%(12/14) (2/14) (0/14) (0/14)	14%-29%-19%-38%(3/21) (6/21) (4/21) (8/21)	<0.0001	50%-21%-21%- 7%(7/14) (3/14) (3/14) (1/14)	22%-33%-33%-11%(2/9) (3/9) (3/9) (1/9)	20%-30%-40%-10%(2/10) (3/10) (4/10) (1/10)	0.833
CD138+ plasma cells	75%- 5%-20%- 0%(15/20) (1/20) (4/20) (0/20)	77%- 0%-23%- 0%(10/13) (0/13) (3/13) (0/13)	80%-10%-10%- 0%(16/20) (2/20) (2/20) (0/20)	0.716	46%-46%-8%- 0%(6/13) (6/13) (1/13) (0/13)	29%-43%-29%- 0%(2/7) (3/7) (2/7) (0/7)	70%-30%- 0%- 0%(7/10) (3/10) (0/10) (0/10)	0.268

^1^Kruskall-Wallis H Test was used for statistical comparison of cell numbers per biopsy area between the three patient groups; Pearson Chi-square test (exact significance, two-sided) was used for comparison of the distribution patterns of the different cell types between the patient groups; ^2^The numbers of evaluated biopsies are given; the numbers of biopsies evaluated for each of the different cell types are given in the pattern of distribution columns. ^3^Mean and SEM of cell numbers per mm ^2^ of biopsy area are given; ^4^The percentage of glomeruli infiltrated by CD68+ macrophages was not significantly different between the patient groups both in 3 months and 1 year protocol biopsies (3 months: 33±7 % (n=20), ABOc; 33±7 % (n=14), ABOi; 21±4 % (n=20), DD; P=0.403; 1 year: 26±7 % (n=11), ABOc; 32±8 % (n=7), ABOi; 19±4 % (n=5), DD; P=0.678); ^5^Distribution patterns of the cell types are given in % of analyzed biopsies for missing or very rare occurrence versus diffuse infiltration versus focal infiltration versus nodular infiltration.

Remarkably, the percentage of interstitial fibrosis/tubular atrophy (IF/TA, given in % of the renal cortex) was significantly increased in the 1-year protocol biopsies of ABOi recipients (26 ± 6% vs. 8 ± 3% in ABOc and 22 ± 4% in DD patients, P = 0.009), and the frequency of nephrosclerosis was also increased in ABOi patients (6/9 (67%) ABOi vs. 2/14 (14%) ABOc and 4/11 (36%) DD patients, P = 0.038).

### 3.8 IgG anti-HLA antibodies

**Figure 3:**
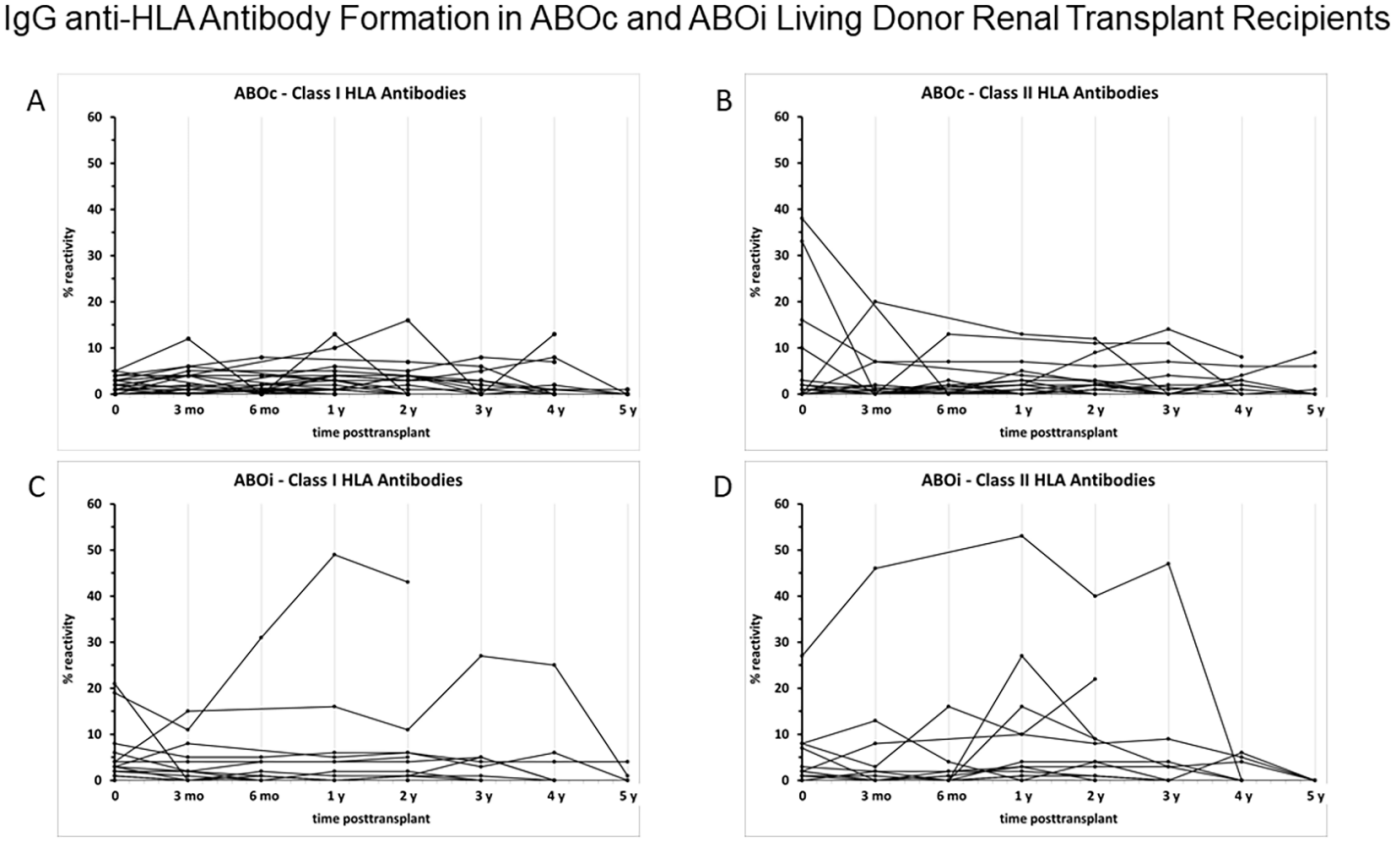
IgG anti-HLA antibody formation in ABOc (**A**: class I; **B**: class II) and ABOi living donor renal transplant recipients (**C**: class I; **D**: class II) pretransplant, 3 and 6 months, and 1,2,3,4 and 5 years posttransplant. IgG anti-HLA antibodies were assessed by single antigen assay on a Luminex basis in ABOi patients (all with rituximab administration pretransplant) and ABOc patients. Three of the ABOc patients received rituximab in the first 4 months postttransplant (protocol violation), two because of donor-specific HLA antibodies (IgG and IgM antibodies, respectively) already pretransplant and one patient because of an acute antibody-mediated rejection 4 months posttransplant. Another ABOc patient received rituximab 6 times because of PTLD starting 43 months posttransplant. Statistical analysis (Mann-Whitney U test) showed no significant differences in HLA antibody formation between ABOc and ABOi recipients with the exception of a clinically not meaningful difference of HLA antibody formation against MHC II antigens 5 years posttransplant (1.0±0.5 %, ABOc versus 0.0±0.0 %, ABOi; P=0.048). No significant differences in HLA antibody formation were found, when patients with early rituximab treatment (all ABOi patients and 3 ABOc patients with treatment pretransplant or up to 4 months posttransplant) were compared with living donor renal transplant recipients without rituximab administration (data not shown).

IgG anti-HLA antibody formation against class I and II antigens was not significantly different between ABOi and ABOc patients up to 5 years posttransplant (**Figure 3**). Also no significant differences in HLA antibody formation were found in peripheral blood when patients with early rituximab treatment (all ABOi patients and 3 ABOc patients with treatment pretransplant or up to 4 months posttransplant) were compared with ABOc recipients without early rituximab administration (data not given). One further ABOc patient received posttransplant rituximab from month 43 on because of PTLD and was therefore not included in the early rituximab group. *De novo* formation of donor-specific HLA antibodies (DSA) occurred in only 1/30 (3%) ABOc and 2/23 (9%) ABOi patients (P = 0.573), whereas pretransplant detectable DSAs (in 6/30 (20%) ABOc vs. 8/25 (32%) ABOi patients; P = 0.363) were lost in 4 of the 6 (67%) ABOc and 7 of the 8 (88%) ABOi patients (P = 0.538), respectively, usually within 6 months posttransplant. Thus, no significant impact of rituximab induction on IgG anti-HLA antibody formation was found, also no impact on *de-novo* HLA antibody formation nor on disappearance of pretransplant HLA antibodies. However, due to the low numbers of *de-novo* DSA formation and pretransplant DSA disappearance, these data do not prove the absence of a relationship.

## 4 Discussion

Newer protocols in blood group incompatible (ABOi) renal transplantation usually use rituximab instead of the previously performed splenectomy pretreatment to suppress anti-ABO titer rebound and anti-ABO associated AMR after ABOi transplantation (10, 11). Rituximab administration was found to improve patient and graft survival compared to pretransplant splenectomy (3, 12). Rituximab has been shown to suppress anti-ABO titer rebound in ABOi renal transplantation. This is also true for suppression of pretransplant anti-ABO titer rebound in so-called high responders, who comprise 10-20% of our patients and do not reach the target anti-ABO titer enabling successful ABOi renal transplantation. We could show that repeated pretransplant rituximab infusions may enable successful ABOi living-donor renal transplantation even in these high responders, without increasing the risk of BK viremia (13). However, an increased risk of severe infectious disease (1–3) and of AMR (2, 3) has been observed after ABOi compared to ABOc renal transplantation. The CTS data (1) described a significantly but only slightly decreased ABOi patient survival in the first posttransplant year only, caused by early severe infectious complications. However, rituximab pretreatment showed no impact on patient survival and also not on death-censored graft survival up to 3 years posttransplant compared to no rituximab treatment. In our current prospective pilot study we confirmed an increased incidence of severe infectious complications, however, only within 2 years posttransplant and not beyond. The incidence of BK viremia in ABOi patients was not significantly elevated, adding to conflicting results in meta-analyses (2, 3). The increased risk of severe infectious complications within 2 years posttransplant in ABOi recipients may be explained by the observed long-term immunological effects after rituximab induction. These comprise downregulated CD4+ and CD8+ T cell counts up to 6 and 3 months, respectively, the profoundly delayed B cell repopulation, most pronounced with regard to memory B cells, and compromised B cell responses found up to 2 years posttransplant. Rituximab-induced B cell elimination together with the observed impaired B cell function may also affect antigen presentation and thereby decrease T-cell activation and infection control. Decreased T cell and CD4+ T cell counts together with impaired CMV-specific T-cell immunity up to 90 days posttransplant has previously been described by Schachtner et al. (14). Thus, antiviral and antifungal prophylaxis is strongly recommended after rituximab induction in ABOi recipients. Pretransplant IVIG is also administered in our center according to the Stockholm and Freiburg ABOi protocols (15, 16). The recommendation of antiviral and antifungal prophylaxis is underlined by data of the Toulouse center (17) showing an increased rate of fungal infections in 77 renal transplant recipients who received rituximab without fungal prophylaxis compared to non-rituximab treated controls. The Hannover transplant center reported an increased incidence of viral infections among 21 consecutive ABOi compared to 47 ABOc renal allograft recipients. ABOi and ABOc patients received CMV prophylaxis in the high risk donor CMV-IgG positive/recipient CMV-IgG negative setting, but no general antiviral prophylaxis with aciclovir or valaciclovir, respectively, or pretransplant IVIG (18).

Meta-analyses of de Weerd and Betjes (2) and Scurt et al. (3) show an increased incidence of AMR in ABOi compared to ABOc patients. Whereas splenectomy patients were involved in the meta-analysis of Scurt et al. (3), de Weerd and Betjes (2) excluded older studies using splenectomy and thus predominantly analyzed rituximab treated ABOi recipients. In contrast to Scurt et al. (3), they also found an increased rate of overall acute rejections in ABOi versus ABOc recipients. Interestingly, in our prospective pilot study with a focus on long-term immunological effects after rituximab induction, we found a low, but significantly increased frequency of AMR in the 5-year follow-up of ABOi versus ABOc and DD recipients, and an increased frequency of overall acute rejections only between 3 and 12 months posttransplant. The lower incidence of acute rejections in the first 3 months posttransplant coincided with the rituximab-induced graft protective effects in regional lymph nodes (decline in naive B cell and short lived plasma cell frequencies) at the time of transplantation and the depletion of B cells in the graft at 3 months posttransplant, whereas evidence of counterregulatory immune activation in 1-year graft biopsies (increase in B cell counts reaching comparable counts with ABOc and DD patients, even enhanced counts of CD3+ T cells, CD68+ macrophages and CD138+ plasma cells) may explain the increased risk of acute rejection between 3 and 12 months posttransplant. The increased AMR frequency in ABOi patients - despite long-term profoundly delayed B cell repopulation together with compromised B cell responses after rituximab induction - may be explained by the missing impact of rituximab on IgG anti-HLA antibody formation, the described immune activation in the graft at 1 year, the increased CD4 helper activity compared to ABOc patients at 2 years when B cell counts were rising, and an increase in NK cell counts 3–5 years posttransplant. Rituximab showed no effect on Tregs in peripheral blood and protocol graft biopsies.

Serum sCD30 and neopterin levels serve as markers of graft outcome. CD30 is an important costimulatory molecule in the regulation of the Th1/Th2 balance and serum sCD30 levels are elevated in diseases with a predominant Th2-type immune response (19). Süsal et al. (20, 21) found that high pretransplant sCD30 levels were associated with impaired kidney graft survival. We showed previously that 1-year serum levels of sCD30 and the T cell/monocyte activation marker neopterin predict graft deterioration by chronic allograft dysfunction (22). In our current study, sCD30 levels in ABOi patients were not significantly elevated compared to ABOc patients, whereas DD patients showed significantly elevated sCD30 levels at 1 year compared to ABOc patients and at 2 years compared to both ABOc and ABOi patients, thereby predicting a worse graft outcome in DD but not in ABOi recipients. Serum neopterin levels were elevated in ABOi compared to ABOc patients at 14 days till 1 year posttransplant, whereas levels in DD versus ABOc patients remained elevated from 3 months to 5 years. These data indicate an increased activation of the T cell/monocyte system up to 1 year posttransplant in ABOi compared to ABOc patients, corresponding to the increased macrophage and T cell infiltration in the 1-year protocol biopsies of ABOi recipients. Overall, evidence of a temporary immune activation in ABOi recipients coincided with an increased AMR incidence and increased interstitial fibrosis/tubular atrophy (IF/TA) in the 1-year protocol biopsies, possibly also a result of the increased acute rejection rate in ABOi patients between 3 and 12 months. It has to be considered, however, that the 1-year protocol biopsy data are less conclusive, as only 41% of the patients were biopsied at 1 year in contrast to 70% at 3 months.

As rituximab induction in ABOi recipients exerts long-term immunosuppressive effects associated with an increased rate of severe infectious complications, but also counterregulatory immune activation and an increased risk of AMR, rituximab-free induction may be an option. Chow et al. (23) used standard immunosuppression without splenectomy or rituximab in 54 unsensitized ABOi recipients and demonstrated a low incidence of 8% (2/25) AMR in 1-year graft biopsies, excellent patient and graft survival and low rates of IF/TA progression. Although these data from Melbourne, Australia, favor a rituximab-free immunosuppression at least in unsensitized ABOi recipients, there is a risk of posttransplant AMR without rituximab induction. Bliesel et al. (24) reported 66 consecutive ABOi transplants in Sidney, Australia, without rituximab induction and found a profoundly increased 39% risk of acute rejection within 3 months with anti-ABO titer rebound in 20%, AMR in 17% of patients, 6 refractory AMR cases requiring splenectomy and 2 early graft losses due to suspected AMR. The historical rituximab-treated ABOi patient group showed only a 6% acute rejection rate and no anti-ABO titer rebound or AMR. In our own series of rituximab-free ABOi living-donor renal transplantation (2016–2024) performed only in patients with baseline anti-ABOi titers of ≤1:16, we experienced an anti-ABO titer rebound with suspected AMR in 1/10 patients which could be completely reversed by 2 rituximab administrations and immunoadsorption (data not published).

The question arises whether the increased risk of severe infectious diseases in rituximab induced ABOi recipients might be avoided by a lower rituximab dosage without increasing the risk of posttransplant anti-ABO titer rebound and AMR. This appears feasible. Lee J et al. (25) found that ABOi recipients induced with a reduced rituximab dosage (200mg absolute dosage) compared with a standard dosage (375mg/m^2^) showed reduced frequencies of severe infectious complications but no significant differences in acute rejection frequencies, patient and graft survival. Moreover, in a report of Masutani et al. (26), 3- and 12-month graft pathology including microvascular inflammation and IF/TA were comparable in a cohort of 101 ABOi recipients after desensitization with low-dose rituximab (200mg) versus 226 ABOc recipients without rituximab. In a review of 21 trials including 1193 ABOi recipients after induction with different dosages of rituximab, Lee HR et al. (27) confirmed the previously mentioned data that low-dose rituximab induction (200mg) lowers the risk of infectious complications without impairing graft outcome. In contrast to our data of a missing effect of rituximab on HLA antibody production, Tomita et al. (28) described a reduced *de-novo* anti-HLA DSA production and reduced acute rejection frequency within 5 years in a propensity score analysis of 318 non-sensitized ABOc living kidney transplants with or without a low-dose rituximab induction (100mg). It has to be discussed, however, whether low-dose rituximab induction is also sufficient in patients with high anti-ABO titers (not analyzed in the review (27)) or even in so-called high responders. Potential differences in long-term immunological effects of low-dose compared to standard-dose rituximab would be of interest.

Our study has limitations. This is a single-center study with a relatively small sample size which compromises the statistical power of the clinical transplant data. However, due to the focus on rituximab-induced long-term immunological effects and the elaborate immunological testing, the analysis of a larger number of patients was not feasible in our center. It is of advantage that all patients were treated with the same maintenance immunosuppression and all ABOi patients received the same desensitization protocol. As there was protocol violation in 4 ABOc and 3 DD patients who received rituximab treatment (pretransplant: ABOc, n=2; DD, n=1), we extended the statistical analysis and evaluated also overall rituximab treated patients and non-treated ABOc and DD patients, respectively. As protocol biopsies were performed in 70% and 41% of patients with functioning grafts at 3 and 12 months, respectively, the 12 month data are less conclusive. Data of *de-novo* HLA antibody formation are not conclusive because of low numbers.

## 5 Conclusions

Our data show that rituximab induction in ABOi recipients is associated with long-term potentially graft protective effects (partial depletion of naive B cells and short lived plasma cells in regional lymph nodes at the time of transplantation, complete B cell depletion in 3-month protocol biopsies, decline of CD4+ and CD8+ T cells and long-term comprised B cell repopulation in peripheral blood, compromised B cell responses) but also a missing impact on HLA antibody formation and even counterregulatory immune activation (recovered B cell counts and even enhanced T cell, macrophage and plasma cell counts in 1-year protocol biopsies, as well as a temporary rise of neopterin, CD4 helper activity and NK cell counts in peripheral blood), going along with an increased risk of acute rejection and especially AMR. Moreover, the long-term immunosuppressive effects of rituximab induction are associated with an increased risk of severe infectious complications. Thus, rituximab induction exerts harmful effects in ABOi patients although it appears to be useful in high-responder patients to enable ABOi transplantation and to suppress posttransplant anti-ABO titer rebound and the associated early AMR. As rituximab-free induction in ABOi recipients appears to carry a risk of anti-ABO titer rebound and AMR, a low dose rituximab induction regimen may provide an option to suppress anti-ABOi titer rebound without an increased risk of infectious complications.

## Data Availability

The datasets presented in this article are not readily available because there is no patient consent for unrestricted transfer of data. Requests to access the datasets should be directed to Prof. Dr. R. Weimer, rolf.weimer@innere.med.uni-giessen.de.

## Glossary

ABOc: ABO blood group compatible
ABOi: ABO blood group incompatible
AIR: acute interstitial rejection
AMR: antibody-mediated rejection
AR: acute rejection
ARF: acute renal failure
ATG: antithymocyte globulin
BKV: BK virus
ClCr: creatinine clearance
CME: continuous medical education
DD: deceased donors
DSA: donor-specific HLA antibodies
HD: hemodialysis
IA: immunoadsorption
IF/TA: interstitial fibrosis/tubulus atrophy
ISC: immunoglobulin-secreting cell
IVIG: intravenous immunoglobulins
LPS: lipopolysaccharide
MEPE: molecules of equivalent phycoerythrin
MHC: major histocompatibility complex
MPGN: membranoproliferative glomerulonephritis
PBMC: peripheral blood mononuclear cells
PD: peritoneal dialysis
PMA: phorbol-12-myristate-13-acetate
PR: preemptive transplantation
PRA max: maximum panel reactive antibodies
PRA rec: recent panel reactive antibodies
PTLD: posttransplant lymphoproliferative disease
RRT: renal replacement therapy
sCD30: soluble CD30
S-Cr: serum creatinine
SEM: standard error of the mean
Tacr: tacrolimus
Tacr-MR: tacrolimus modified-release
